# Selectivity in subunit composition of Ena/VASP tetramers

**DOI:** 10.1042/BSR20150149

**Published:** 2015-09-10

**Authors:** Daisy N. Riquelme, Aaron S. Meyer, Melanie Barzik, Amy Keating, Frank B. Gertler

**Affiliations:** * The David H. Koch Institute for Integrative Cancer Research, Massachusetts Institute for Technology, Cambridge, MA 02139, U.S.A.; † Department of Biology, Massachusetts Institute of Technology, Cambridge, MA 02139, U.S.A.; § Department of Biological Engineering, Massachusetts Institute of Technology, Cambridge, MA 02139, U.S.A.

**Keywords:** actin, Ena/VASP, EVH2, EVL, Mena, oligomerization, VASP

## Abstract

The members of the actin regulatory family of Ena/VASP proteins form stable tetramers. The vertebrate members of the Ena/VASP family, VASP, Mena and EVL, have many overlapping properties and expression patterns, but functional and regulatory differences between paralogues have been observed. The formation of mixed oligomers may serve a regulatory role to refine Ena/VASP activity. While it has been assumed that family members can form mixed oligomers, this possibility has not been investigated systematically. Using cells expressing controlled combinations of VASP, Mena and EVL, we evaluated the composition of Ena/VASP oligomers and found that VASP forms oligomers without apparent bias with itself, Mena or EVL. However, Mena and EVL showed only weak hetero-oligomerization, suggesting specificity in the association of Ena/VASP family members. Co-expression of VASP increased the ability of Mena and EVL to form mixed oligomers. Additionally, we found that the tetramerization domain (TD) at the C-termini of Ena/VASP proteins conferred the observed selectivity. Finally, we demonstrate that replacement of the TD with a synthetic tetramerizing coiled coil sequence supports homo-oligomerization and normal VASP subcellular localization.

## Abstract (precis)

Ena/VASP tetramer composition was analysed and mixed oligomerization of Mena with EVL was found to be unfavourable, while other paralogue combinations formed without apparent bias. The tetramerization domain of Ena/VASP proteins is responsible for their selective tetramer formation.

## INTRODUCTION

The Ena/VASP family comprises a group of highly conserved proteins involved in the regulation of cell morphology, adhesion and motility [1–4]. Ena/VASP proteins localize to actin-rich structures, including sites of cell–cell and cell–matrix adhesions, the edge of lamellipodia and the tips of filopodia [1,4–6]. The family members play important roles in diverse cell types, including fibroblasts [7], endothelial cells [8], platelets [9], epithelial cells [4] and neurons [3]. Vertebrates express three closely related Ena/VASP family members, Mena, VASP and EVL. While mice lacking any one of the three are viable and exhibit only subtle defects, combined deficiency in Mena, VASP and EVL results in embryonic lethality, accompanied by a variety of morphogenic and cardiovascular defects [3,6,8–11].

**Figure 1:**
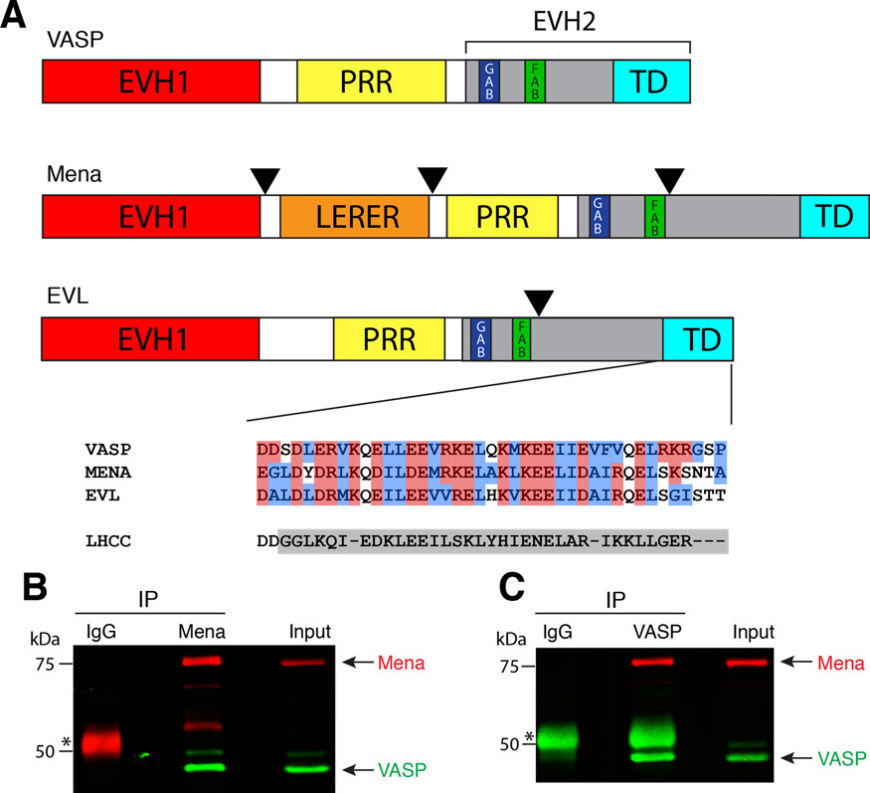
Endogenous Mena:VASP protein complexes are detected *in vivo* (**A**) Schematic of Ena/VASP family domain structure. Within the EVH2 domain (grey) are the G-actin and F-actin binding motifs (GAB and FAB, respectively) and the tetramerization domain (TD). Arrowheads denote sites of alternative exon inclusion. The alignment of the TDs of the mouse Ena/VASP proteins is shown. Sequences are highlighted according to residue properties (red=acidic or basic, blue=hydrophobic). For VASP^LHCC^, the TD of VASP was replaced with an unrelated synthetic sequence that forms a left-handed tetrameric coiled coil, highlighted in grey. Mena (**B**) or VASP (**C**) was immunoprecipitated (IP) from Rat2 (fibroblast cell line) lysates using αMena monoclonal antibody or αVASP polyclonal antibody, respectively. Precipitated proteins were analysed by western blots probed with αMena antibody (red) or αVASP antibody (green). Control IPs were performed in parallel using mouse (**B**) or rabbit (**C**) IgG. Asterisks indicate IgG heavy chains reacting with detecting antibody. Input is 10% of IP. Mena and VASP blots shown are representative of three (Mena IP) or two (VASP IP) independent experiments.

Ena/VASP proteins possess a modular domain organization, consisting of two Ena/VASP homologous regions (EVH1 and EVH2) and a proline-rich region (PRR) of varying length (Figure 1A). The EVH1 domain mediates interactions between Ena/VASP members and proteins containing the highly conserved poly-proline core consensus motif ‘FPPPP’ (FP4) [12,13]. In the centre of the protein, the PRR possesses binding sites for SH3 and WW-domain containing proteins and the actin-monomer binding protein, profilin [14–16]. The C-terminal EVH2 domain consists of G-actin and F-actin binding sites, termed GAB and FAB regions, respectively [17–19]. At the most C-terminal portion of the EVH2 domain, a highly conserved tetramerization domain (TD) mediates the formation of Ena/VASP tetramers [14,18–20]. Both full length VASP and the isolated EVH2 domain form stable tetramers as indicated by analytical centrifugation [14,19,21]. The TD of VASP has been crystalized and shown to form a right-handed, coiled-coil tetramer [34].

Ena/VASP proteins promote actin polymerization by increasing the elongation rate of actin filaments and by protecting barbed ends from capping proteins, thus delaying termination of filament growth [7,14,17,21]. The importance of this activity has been demonstrated in different cellular contexts. In fibroblasts, for example, loss of Ena/VASP proteins results in the formation of lamellipodia with short, highly branched actin filament networks, while overexpression of Ena/VASP proteins leads to longer, less branched actin networks [7]. Ena/VASP family members are also key regulators of the formation and elongation of filopodia, structures which consist of long, parallel bundles of F-actin [22–24].

A number of Ena/VASP functions are critically dependent upon their ability to tetramerize [14,25]. For example, deletion of the TD ablates Ena/VASP-dependent filopodia formation and elongation [24]. In experiments where the TD of VASP was replaced with a dimerization sequence, artificial VASP dimers were compromised in their ability to promote actin elongation, supporting a model in which higher order oligomers are critical for full Ena/VASP protein function [21].

Cells may express more than one Ena/VASP paralogue, and it has been suggested that Mena, VASP and EVL can form mixed tetramers [1,26]. The three paralogues differ in their ability to promote actin polymerization *in vitro*, and additional evidence suggest paralogue-specific interactions and modes of regulation [27–30]. These findings raise the intriguing possibility that Ena/VASP hetero-tetramer formation could provide a means to fine-tune the actin-polymerization activity of Ena/VASP tetramers, as well as to increase the diversity of their interactions and regulation. The ability of Ena/VASP proteins to form oligomers consisting of multiple Ena/VASP family members has been demonstrated *in vitro* [26,31], but the extent to which homo- versus hetero-tetramerization occurs *in vivo* has not been addressed systematically.

In this study, we evaluate the composition of mammalian Ena/VASP multimers in cells. We confirm that mixed Ena/VASP oligomers are formed, but, unexpectedly, find that particular subunit combinations are disfavoured. Furthermore, we demonstrate that the TD controls the selectivity of mixed Ena/VASP tetramer formation. Hetero-tetramerization may provide a mechanism to fine-tune Ena/VASP function and regulation.

## RESULTS

### Mena and VASP associate *in vivo*

We sought to determine the extent to which Ena/VASP paralogues hetero-oligomerize in cells. To confirm that members of the Ena/VASP family form mixed complexes *in vivo*, the association of endogenous Mena and VASP was determined in Rat2 fibroblasts, which express both Mena and VASP at readily detectable levels (Figure 1) [2]. Endogenous Mena was immunoprecipitated from Rat2 lysates and endogenous VASP was found to co-immunoprecipitate (co-IP) (Figure 1B). Reciprocally, immunoprecipitation of endogenous VASP confirmed the observed association with Mena (Figure 1C). Control immunoprecipitation with isotype-matched antibodies demonstrated the specificity of the Mena:VASP co-IP. Thus, endogenous Mena and VASP can be detected in complexes isolated from cells.

It is possible that mixed complexes could arise from the dissociation and reassembly of oligomers during cell lysis and subsequent immunoprecipitation. To address this, MV^D7^ cells, a mouse embryonic fibroblast cell line derived from mice genetically null for Mena and VASP and lacking detectable EVL expression [2], were transfected with either EGFP-Mena or EGFP-VASP alone. Cells were mixed at a 1:1 ratio of VASP transfected cells to Mena transfected cells, and Mena was immunoprecipitated from lysates as above. VASP did not co-IP with Mena in this assay (Supplementary Figure S1A), confirming that oligomers assembled within cells are stable under the biochemical isolation conditions employed here. This is consistent with structural and biochemical data indicating that VASP homo-tetramers are extremely stable [14,19].

### Mena:VASP complex assembly is mediated by the TD

**Figure 2:**
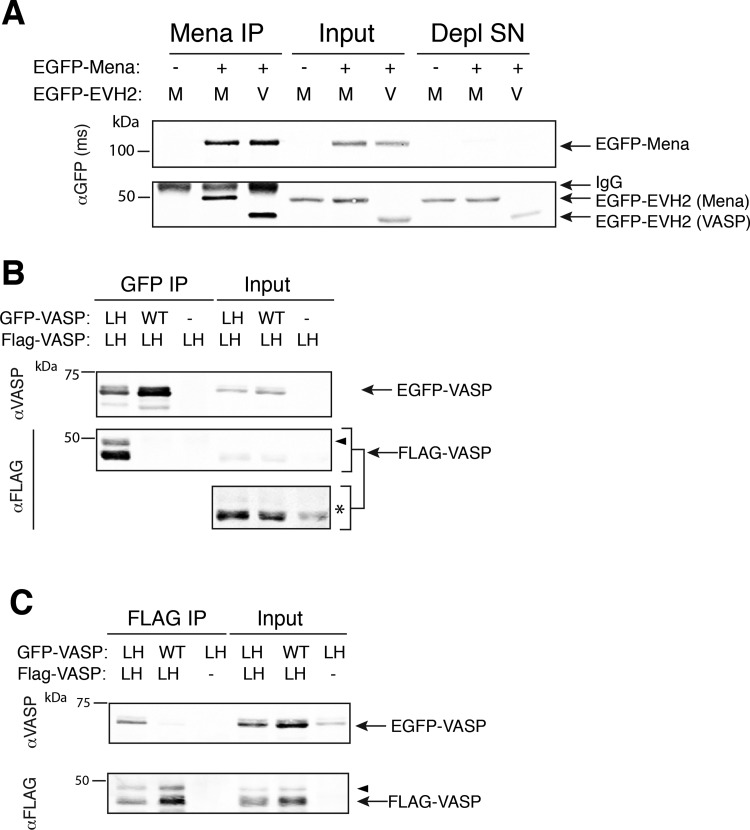
TD mediates oligomerization of Ena/VASP proteins (**A**) MV^D7^ cells were co-transfected with EGFP-Mena and the EVH2 domains of Mena (‘M’) or VASP (‘V’) fused to EGFP. EGFP-Mena was immunoprecipitated with αMena antibody and interactions with EVH2 domains were detected with αGFP antibody. Input is 10% of IP (‘Input’) and αMena-depleted supernatant (‘Depl SN’) indicates near complete immuno-depletion of Mena from lysates. The EVH2 domain of Mena is larger than the EVH2 domain of VASP, and thus migrates at a higher molecular weight. Blots shown are representative of two independent experiments. (**B**) and (**C**), cells were co-transfected with FLAG-VASP^LHCC^ (‘LH’) and either EGFP-VASP^WT^ (‘WT’) or EGFP-VASP^LHCC^ (‘LH’). αEGFP (**B**) or αFLAG (**C**) antibodies were used to immunoprecipitate tagged proteins. Western blots were probed with αVASP and αFLAG antibody, as indicated. Contrast-enhanced image of input for (**B**) indicated by asterisk. Input is 5% of IP. Blots shown in (**B**) and (**C**) are representative of three independent experiments each. Arrowheads denote phosphorylated VASP, which induces a well-characterized electrophoretic mobility shift.

Ena/VASP proteins interact with a number of shared binding partners. Some proteins, such as zyxin [32] and Lpd [33], can bind multiple Ena/VASP proteins simultaneously. These interactions are mediated via the EVH1 domain of Ena/VASP proteins [13]. To eliminate the possibility that the associations observed between Mena and VASP occur as a result of potential scaffolding effects through shared EVH1-binding partners (e.g. zyxin), or through interactions mediated by the PRR, we tested whether we could observe isolated EVH2 domains in complex with EGFP-Mena. We co-transfected EGFP-Mena with the EVH2 domain of Mena or VASP fused to EGFP into MV^D7^ cells. Both EVH2 domains associated with immunoprecipitated EGFP-Mena (Figure 2A), in agreement with previous studies demonstrating that the EVH2 domain is sufficient for oligomerization [18,19,31,34].

VASP and *Drosophila* Ena have each been demonstrated to form tetramers [14,19–21]. Given the structural data showing that the VASP TD is a tetramer [34] and that the TD sequence is highly conserved among Ena/VASP proteins, it is likely that Ena/VASP proteins generally form tetramers. To verify that the EVH2 domain of Mena forms tetramers, lysates from MV^D7^ cells expressing EGFP-EVH2 (from Mena) were treated with a chemical crosslinking agent and analysed for mobility by SDS/PAGE followed by western blotting. The cross-linked EGFP-EVH2 migrated with an apparent molecular weight of ∼200 kDa while the EGFP-EVH2 from untreated lysates migrated at ∼50 kDa, consistent with idea that, like VASP, the Mena EVH2 domain forms tetramers (Supplementary Figure S1B).

The EVH2 domain contains the GAB and FAB regions, which bind to G- and F-actin, respectively [35], raising the possibility that Ena/VASP complexes may form via scaffolding interactions mediated by actin. To test if actin binding is sufficient to mediate mixed Ena/VASP complexes, we engineered a version of VASP to force VASP homo-tetramer formation independently of the conserved TD, and assayed for the formation of mixed Ena/VASP complexes by co-IP. The designed synthetic tetramer sequence of GCN4 p-LI was based on the coiled-coil oligomerization domain of yeast transcription factor GCN4, a well-studied model of oligomerization [36,37]. GCN4 p-LI d has previously been shown to function modularly in replacement of the TD of the pore protein KcsA [38] and of p53 [39]. Since the VASP TD forms a parallel, right-handed, tetrameric coiled coil [34], while the synthetic TD forms a left-handed, tetrameric coiled coil, we refer to the chimeric VASP as VASP^LHCC^ (Figure 1A).

We assayed if interactions with actin or other scaffolding proteins were sufficient for VASP^LHCC^ to associate with wild-type VASP (VASP^WT^). VASP^LHCC^ and VASP^WT^ were expressed at comparable levels (Figure 2B, input), suggesting that the expression and stability of VASP protein was not grossly altered by substitution of the synthetic coiled coil for the TD. To test whether VASP^LHCC^ could self-associate as well as associate with VASP^WT^, we performed co-IP experiments using lysates of MV^D7^ cells expressing FLAG-VASP^LHCC^ along with either EGFP-VASP^WT^ or EGFP-VASP^LHCC^. FLAG-VASP^LHCC^ was readily detected to co-IP with EGFP-VASP^LHCC^, but not with EGFP-VASP^WT^ (Figure 2B). Reciprocally, EGFP-VASP^WT^ failed to associate with immunoprecipitated FLAG-VASP^LHCC^, whereas EGFP-VASP^LHCC^ did co-IP with FLAG-VASP^LHCC^ (Figure 2C). Thus, the left-handed VASP^LHCC^ forms oligomers only with VASP^LHCC^, and not with wild-type Ena/VASP proteins. Importantly, these data also confirm that the observed Ena/VASP oligomerization is dependent upon the TD, and that actin or other binding partners are not sufficient to mediate Ena/VASP oligomerization.

### Mena:VASP association is consistent with random tetramer oligomerization

**Figure 3:**
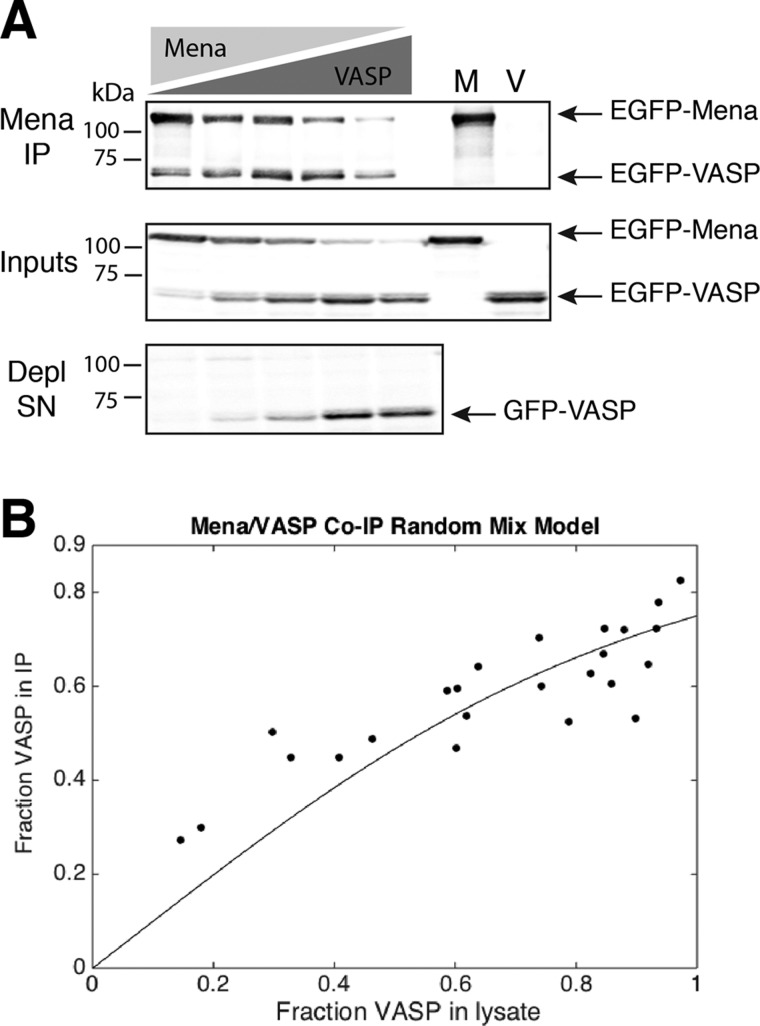
Mena and VASP oligomerization is consistent with random tetramerization (**A**) EGFP-tagged Mena and VASP were co-expressed in MV^D7^ fibroblasts at varying ratios, or Mena (‘M’) or VASP (‘V’) alone. Mena was immunoprecipitated from lysates and interacting VASP was detected via the shared EGFP tag using an αGFP antibody (top blot). Protein lysates prior to IP (‘Input’) and of αMena-depleted supernatant (‘Depl SN’) (middle and bottom blots, respectively) indicate near complete immuno-depletion of Mena from lysates. Bands were quantified by densitometry and were used to calculate the VASP fraction of total Ena/VASP protein in Mena IPs and in lysates. Blots shown are representative of five independent experiments. (**B**) The VASP fraction of total Ena/VASP protein in lysates (*x*-axis) was plotted against the VASP fraction of total Ena/VASP protein in Mena IPs (*y*-axis). The curved line indicates expected results based on a model of randomly formed tetramers. Each point represents an independent Mena IP.

While the co-IP of Mena with VASP in Rat2 cells indicated that endogenous Mena and VASP form mixed complexes within cells (Figure 1), it did not provide information on the composition of the observed oligomers. We wondered if Ena/VASP tetramers would exhibit bias in the formation of particular subunit compositions, such as a preference to form homo-oligomers over hetero-oligomers. We performed a series of co-IP experiments to quantify the abundance of Mena and VASP in mixed oligomers. To control the relative expression of Ena/VASP proteins, we transfected varying amounts of EGFP-Mena and EGFP-VASP into MV^D7^ cells. Co-transfected cells were lysed and Mena was immunoprecipitated from the lysates using an αMena monoclonal antibody known to recognize an epitope absent in VASP [23]. As expected, control experiments demonstrated that the αMena antibody showed no detectable cross-reactivity with EGFP-VASP (Figure 3A).

Our experimental design allowed us to compare the relative levels of EGFP-VASP and EGFP-Mena expressed in cell lysates or in αMena immunoprecipitates directly by western blot, using an antibody against the shared GFP tag. The large molecular weight difference between EGFP-Mena (∼100 kDa) and EGFP-VASP (∼80 kDa) allowed unambiguous identification of each species (Figure 3A). Band intensities were quantified to calculate the relative composition of Ena/VASP proteins present in immunoprecipitated complexes and the fraction present in cell lysates [EGFP-VASP/(EGFP-Mena+EGFP-VASP)] across a range of expression levels of the two proteins. The resulting data were plotted and fitted to a model in which tetramer formation between Mena and VASP monomers is assumed to be random (Figure 3B). The model captured our measured associations (*r*^2^=0.76), consistent with random association largely determining the amount of Mena:VASP hetero-oligomerization. Additionally, these data are consistent with the assumption that the immunoprecipitated complexes are likely Ena/VASP tetramers. These observations suggest that Mena and VASP can randomly associate as hetero-tetramers.

**Figure 4:**
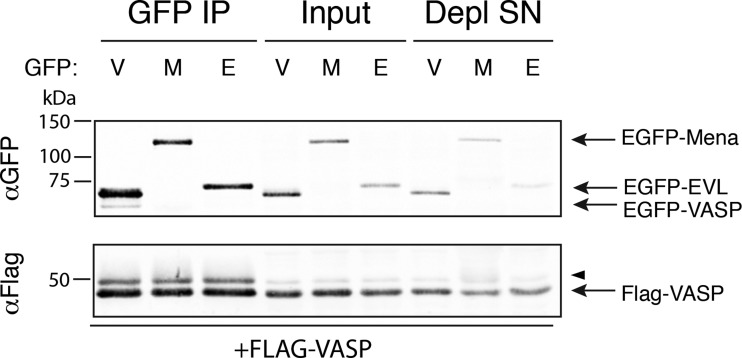
VASP oligomerizes with itself, Mena and EVL without bias MV^D7^ cells were co-transfected with FLAG-VASP and either EGFP-VASP (‘V’), EGFP-Mena (‘M’) or EGFP-EVL (‘E’). EGFP-tagged Ena/VASP proteins were immunoprecipitated from lysates and interacting FLAG-VASP detected with αFLAG antibody. Phosphorylation of VASP induced a well-characterized electrophoretic mobility shift of VASP and was observed in the IP lanes, indicated by an arrowhead. Input is 5% of IP. Immuno-depleted supernatants (‘Depl SN’) illustrate GFP IP captured a significant fraction of EGFP-tagged Ena/VASP proteins expressed. Blots shown are representative of two independent experiments.

In addition to examining VASP:Mena interactions, we wanted to determine if VASP and EVL mixed tetramers also assemble without bias. To test this, FLAG-VASP was co-transfected into MV^D7^ cells with EGFP-VASP, EGFP-Mena or EGFP-EVL. Immunoprecipitation of the EGFP-tagged Ena/VASP proteins using a polyclonal GFP antibody demonstrated that VASP:VASP complexes occurred to a similar extent as VASP:Mena and VASP:EVL complexes (Figure 4). These results suggest that VASP forms mixed oligomers with all Ena/VASP proteins expressed in a cell with little to no compositional bias.

### Mena preferentially oligomerizes with VASP over EVL

Mena can be alternatively spliced to create isoforms which all share the same TD, including Mena11a [40] and Mena^INV^ [1,27]. The various Mena protein isoforms have distinct functions compared to Mena lacking these additional sequences (Mena^classic^) [27,41]. Of particular interest in this context, the 11a sequence, when included, is found within the EVH2 domain, raising the possibility that it may influence tetramer formation due to its proximity to the TD. We tested whether all Mena isoforms could form mixed tetramers by co-IP. To determine if 11a or INV inclusion altered the ability of Mena to homo-oligomerize with Mena^classic^, MV^D7^ cells were co-transfected with FLAG-Mena^classic^ and either EGFP-Mena^INV^, EGFP-Mena11a, or EGFP-Mena^classic^. Immunoprecipitation experiments using a GFP polyclonal antibody indicated that FLAG-Mena^classic^ bound all Mena isoforms to a similar extent (Supplementary Figure S2). This suggests that oligomers consisting of mixed Ena/VASP isoforms can exist in cells expressing multiple Mena isoforms.

**Figure 5:**
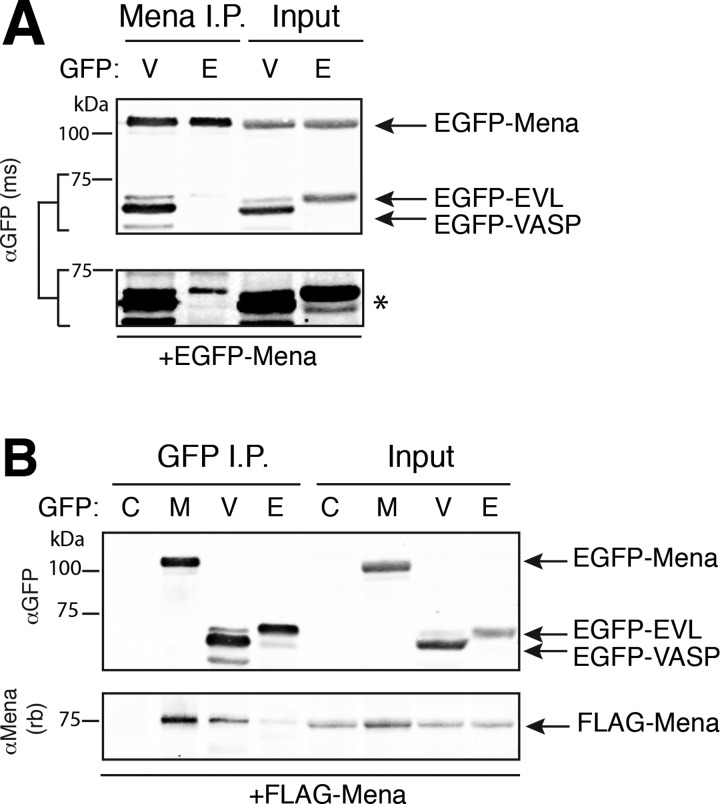
Mena and EVL association is disfavoured (**A**) EGFP-Mena was immunoprecipitated from lysates of MV^D7^ cells co-transfected with EGFP-Mena and either EGFP-VASP (‘V’) or EGFP-EVL (‘E’). Interacting Ena/VASP proteins were detected by immunoblot with αGFP antibody. A weak co-IP of EGFP-EVL can only be detected by contrast enhancement of the bracketed portion (*, bottom panel). Input is 10% of IP. (**B**) EGFP-tagged constructs were immunoprecipitated from MV^D7^ fibroblasts co-transfected with FLAG-Mena and control EGFP (‘C’), EGFP-Mena (‘M’), EGFP-VASP (‘V’) or EGFP-EVL (‘E’). Interacting FLAG-Mena was detected using a polyclonal αMena antibody. Input is 10% of IP. Blots shown are representative of three or two independent experiments for (**A**) and (**B**), respectively.

As there appeared to be no bias in oligomer formation between VASP and either Mena or EVL, we tested whether all Ena/VASP proteins can form mixed tetramers by examining the remaining pairwise combinations. To evaluate oligomerization of Mena with EVL, we performed co-IP experiments of EGFP-tagged Ena/VASP proteins (as above) to compare the formation of Mena:EVL complexes to the formation of Mena:VASP complexes. To our surprise, relative to EGFP-VASP, we found only a very weak association of EGFP-EVL with EGFP-Mena (Figure 5A).

To confirm this result, FLAG-Mena was co-transfected with EGFP, EGFP-Mena, EGFP-VASP or EGFP-EVL in MV^D7^ fibroblasts. FLAG-Mena associated with immunoprecipitated EGFP-Mena and EGFP-VASP, but not with EGFP-EVL or control EGFP (Figure 5B). Together, these results suggest that Mena can only weakly associate with EVL, but oligomerizes readily with VASP and itself.

### Ena/VASP TD domain confers the selectivity of hetero-tetramer formation

**Figure 6:**
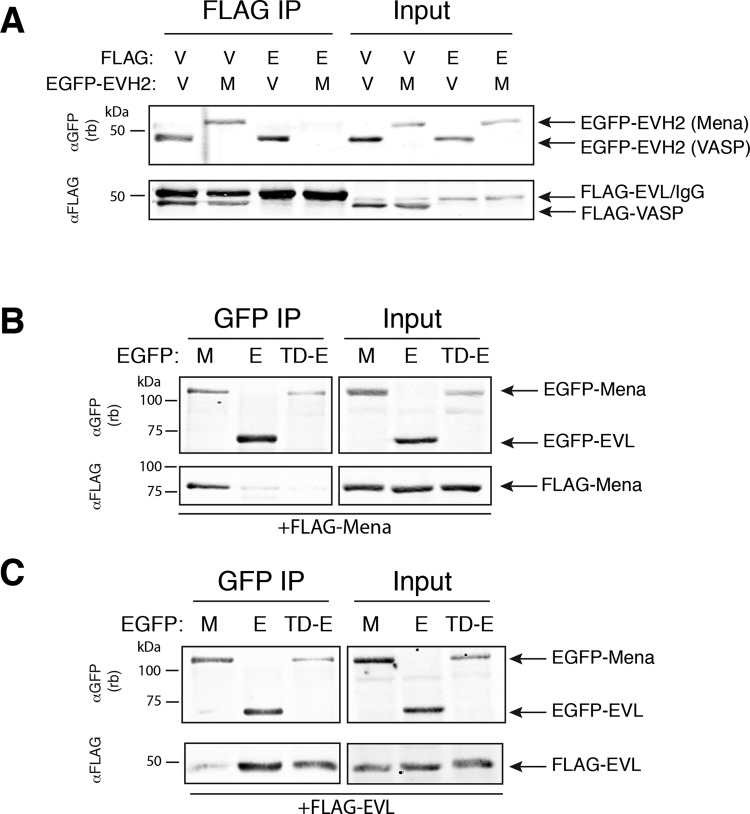
TD specifies composition of Ena/VASP oligomers (**A**) FLAG-tagged proteins were immunoprecipitated from MV^D7^ cells co-transfected with Flag-VASP (‘V’) or Flag-EVL (‘E’) and the EGFP-EVH2 domains of VASP (‘V’) or Mena (‘M’). EGFP-EVH2 domain interactions were detected using an αGFP antibody. FLAG-EVL co-migrates with IgG heavy chain, as indicated. Input is 5% of IP. Blot shown is representative of two independent experiments. MV^D7^ fibroblasts were co-transfected with FLAG-VASP (**B**) or FLAG-EVL (**C**) and either EGFP-Mena (‘M’), EGFP-VASP (‘V’), or chimeric EGFP-Mena^TD-EVL^ (‘TD-E’). EGFP-tagged proteins were immunoprecipitated from lysates and detected with αGFP antibody and interacting FLAG-tagged proteins were detected with αFLAG-M2 antibody. Input is 5% of IP. Blots shown are representative of three independent experiments each for (**B**) and (**C**).

We hypothesized that the weak association of Mena with EVL, relative to Mena (or EVL) with VASP, may arise from differences within their EVH2 domains. Therefore, we tested the ability of EVL to associate with the EVH2 domains of VASP and Mena by co-IP. As a positive control, we found that the EVH2 domains of both Mena and VASP were readily detected to co-IP with Flag-VASP, as expected. In contrast, the EVH2 domain of VASP, but not of Mena, was found to co-IP with FLAG-EVL (Figure 6A). Hence, the EVH2 domain (containing the TD) dictates the specificity of Ena/VASP oligomer formation.

The TD in the EVH2 domain of Ena/VASP proteins mediates tetramerization [26,31,34], but it is uncertain whether differences between the TD of Mena and EVL would account for the observed specificity in Ena/VASP oligomer composition. To test whether the TD directs selectivity of tetramer composition, we generated a chimeric EGFP-Mena construct (Mena^TD-EVL^), in which the TD of Mena was replaced with the homologous sequence of EVL (Figure 1A). MV^D7^ cells were co-transfected with EGFP-Mena, EGFP-EVL or EGFP-Mena^TD-EVL^ and either FLAG-Mena or FLAG-EVL. EGFP-tagged proteins were immunoprecipitated and the associated FLAG-tagged proteins were detected by western blot. In agreement with earlier observations (Figure 5), FLAG-Mena associated with EGFP-Mena but not EGFP-EVL (Figure 6B), while FLAG-EVL associated with EGFP-EVL but not EGFP-Mena (Figure 6C). However, chimeric Mena^TD-EVL^ behaved similarly to EGFP-EVL and not EGFP-Mena: it associated robustly with FLAG-EVL, but not with FLAG-Mena. These observations confirm that the TD is sufficient to mediate the selectivity of tetramer assembly of Ena/VASP proteins.

### VASP enhances the association of EVL and Mena

The experiments performed thus far assayed the ability of pairwise combinations of Ena/VASP proteins to form heteromeric complexes. However, some cell types express all three Ena/VASP paralogues [29,42]. Given the bias against mixed Mena:EVL oligomers, we wondered whether hetero-tetramers containing all three Ena/VASP family members could form. We hypothesized two potential scenarios for mixed tetramer formation in cells expressing all three paralogues: (1) oligomers containing all three Ena/VASP proteins form, with VASP supporting inclusion of both Mena and EVL, or (2) assembled oligomers form distinct sets of tetramers consisting only of EVL and VASP, or Mena and VASP, but not all three.

**Figure 7:**
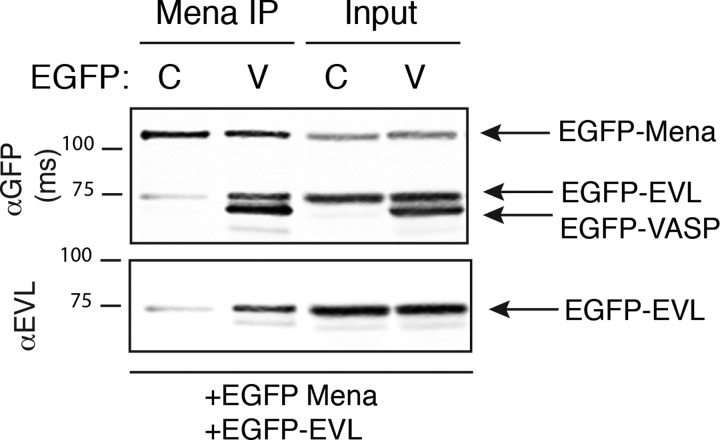
VASP enhances Mena:EVL oligomerization Mena was immunoprecipitated from MV^D7^ fibroblasts co-transfected with EGFP-Mena, EGFP-EVL, and either control EGFP (‘C’) or EGFP-VASP (‘V’). EGFP-tagged proteins were detected with αGFP antibody and EVL was detected using αEVL polyclonal antibody. Blots shown are representative of two independent experiments.

MV^D7^ cells were transfected with EGFP-Mena, EGFP-EVL, and either EGFP or EGFP-VASP. We found that co-expression of EGFP-VASP increased the amount of EVL in complex with immunoprecipitated Mena, compared with EGFP alone (Figure 7). This suggests that Ena/VASP proteins can form oligomers containing all three family members, with VASP acting as a bridge to promote the association of Mena and EVL.

### VASP^LHCC^ localization is similar to VASP^WT^

**Figure 8:**
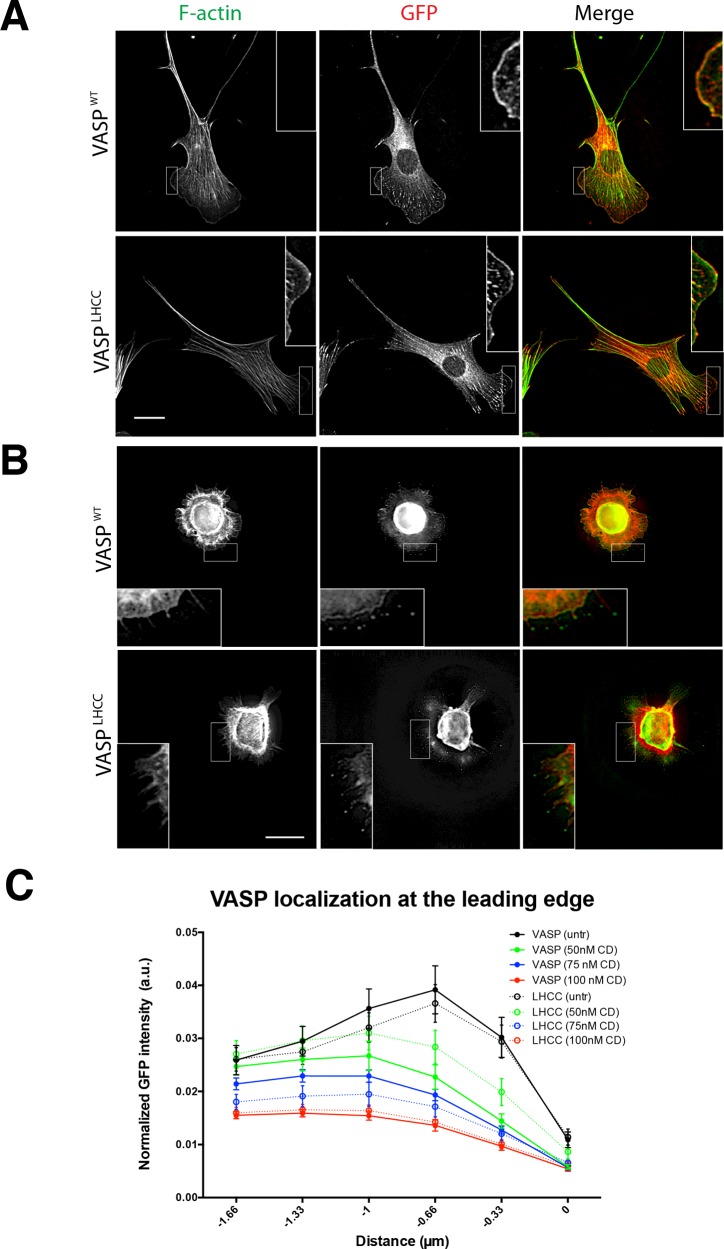
VASP^LHCC^ localization is similar to VASP^WT^ MV^D7^ fibroblasts stably expressing EGFP-VASP^WT^ or EGFP-VASP^LHCC^ were fixed and imaged by immunofluorescence. F-actin was visualized with fluorescent phalloidin. (**A**) EGFP-VASP^WT^ and EGFP-VASP^LHCC^ both localize to focal adhesions and to the leading edge (inset) in cells fixed 24 h after plating. Scale bar, 20 μm. (**B**) EGFP-VASP^WT^ and EGFP-VASP^LHCC^ both localize to filopodial tips (inset) extending from spreading cells fixed 20 min after plating. Scale bar, 20 μm. (**C**) MV^D7^ cells expressing EGFP-VASP^WT^ or EGFP-VASP^LHCC^ plated on fibronectin-coated coverslips were fixed and stained after incubation with the indicated concentration of CD. GFP intensities at cell edges were measured and normalized to the total GFP intensity of the cell. The mean (+/-S.E.M) of the normalized measurements of at least 20 cells are plotted. The edge of the cell is represented by distance 0 on the *x*-axis and negative values indicate intracellular distances.

It has been suggested that the right-handedness of the TD's coiled coil supports Ena/VASP binding to the barbed ends of actin filaments, which also form right-handed coils [34]. Deletion of the TD alters the localization of Ena/VASP proteins [25,42]. We examined the localization of EGFP-VASP^LHCC^ to determine whether the synthetic tetramer can support subcellular targeting of VASP similar to that of the wild-type TD. Using MV^D7^ cells stably expressing EGFP-VASP^WT^ or EGFP-VASP^LHCC^, we found that EGFP-VASP^LHCC^ localized to focal adhesions (Figure 8A) and to the edge of lamellipodia in MV^D7^ cells (Figure 8A, inset), similarly to EGFP-VASP.

A subpopulation of MV^D7^ cells extends filopodia while spreading on fibronectin [24]. To determine if VASP^LHCC^ localizes to the tips of filopodia, spreading MV^D7^ cells stably expressing either EGFP-VASP^WT^ or EGFP-VASP^LHCC^ were plated on fibronectin-coated coverslips. In cells spreading in the characteristic filopodial mode, both VASP^WT^ and VASP^LHCC^ were concentrated at the tips of filopodial protrusions (Figure 8B, inset). These observations suggest that substitution of the TD with a synthetic tetramerizing sequence does not disrupt VASP localization.

In fibroblasts, accumulation of wild-type Ena/VASP proteins at the leading edge of protruding lamellipodia depends, in part, upon binding to F-actin barbed ends [4,7,25,33]. Treatment of cells with a low dose (25–150 nM) of Cytochalasin D (CD) displaces Ena/VASP proteins from the leading edge by binding actin barbed ends, making them unavailable to Ena/VASP proteins [4,7,33]. To assess the role of handedness in the efficiency of barbed end-dependent localization of VASP to lamellipodial edges, we compared the localization VASP^LHCC^ and VASP^WT^ in cells after treatment with a range of CD concentrations (50–100 nM). Lamellipodin (Lpd) staining identified actively protruding lamellipodia. As expected, the low-dose CD treatments did not grossly disrupt the actin cytoskeleton (Supplementary Figure S3) [7] or displace the lamellipodial protein Lpd [33]. The fluorescence intensity of VASP at the leading edge of Lpd-positive protrusions was measured and normalized to total EGFP-VASP fluorescence intensity. At each concentration of CD used, both VASP variants were gradually displaced from the cell edge to similar degrees. No statistically significant differences were observed between the localization of VASP^WT^ and VASP^LHCC^ at the leading edge (Figure 8C). These observations suggest that the handedness of VASP's TD is not essential for its association with free barbed ends, at least to the extent that this interaction contributes to VASP localization at the lamellipodial edge. Thus, the sole function of the TD of Ena/VASP proteins appears to be the mediation of the selective formation of Ena/VASP homo- and hetero-tetramers.

## DISCUSSION

It had been postulated that Ena/VASP family members assemble randomly into oligomers [26,43]. Our results demonstrate that although Ena/VASP proteins can form mixed oligomers, the composition of these complexes is biased against certain paralogue combinations. While VASP freely forms oligomers with all mammalian Ena/VASP paralogues, there is a bias against the formation of Mena:EVL hetero-tetramers. Additionally, we demonstrated that the TD of Ena/VASP proteins governs this selectivity. Scaffolding via actin or other binding partners does not play a dominant role in Ena/VASP oligomerization, as substituting the right-handed TD in VASP with a left-handed TD allowed only for homo-oligomerization. Furthermore, substitution of the TD does not grossly affect VASP localization or its ability to concentrate at the leading edge of lamellipodia, suggesting that the handedness of the coiled coil is not critical for this F-actin barbed-end-dependent component of Ena/VASP localization.

### The EVH2 domain mediates selective formation of tetramers

Although co-IP of Mena and VASP has been detected previously [1], this is the first study to systematically assess hetero-oligomerization of Mena, VASP and EVL in cells. Our data demonstrate that hetero-oligomeric complexes between Ena/VASP family members form in cells and that oligomerization of Mena and VASP is consistent with random tetramerization. Co-IP experiments of EVL with the EVH2 domains of VASP and Mena confirmed that the EVH2 domain mediates multimerization [18,19] and also demonstrated that the EVH2 domain contains sequences that confer selectivity of oligomer formation. We found that substitution of the TD of Mena with the TD of EVL inverted Mena's bias against binding EVL. Chimeric Mena^TD-EVL^ preferentially bound EVL but did not associate with wild-type Mena. Therefore, we conclude that residues within the TDs of Mena, VASP and EVL specify their interactions.

Future studies are necessary to shed light on the critical residues involved in specifying the composition of hetero-tetramers. Despite the high degree of similarity between the TDs of Ena/VASP proteins [1,18,44] (Figure 1A), potential side-chain interactions between residues of Mena and EVL may inhibit their association. Currently, only the TD of VASP homo-tetramers has been crystalized [34]. Structural and mutational analyses of the TD of Mena and EVL homo-tetramers and of Ena/VASP hetero-tetramers are necessary to determine which residues may inhibit the association between the TDs of Mena and EVL.

### Potential effects of Ena/VASP hetero-tetramerization on actin dynamics

Ena/VASP proteins bind polymerizing actin barbed ends and facilitate the addition of actin monomers. The PRR region and adjacent GAB bind profilin–actin in a ternary complex, which facilitates the transfer of the actin monomer on to the growing filament [15,44,45]. Recent data determined that changes in the relative levels of profilin influence the architecture of the actin structures and alter the behaviour of both normal and cancer cells [29,54]. There are multiple profilin isoforms in mammalian cells and differences in the function and biochemical properties have been observed between isoforms [29,55]. Previously, it was shown that profilins enhance Ena/VASP mediated actin-polymerization rates [14,20]. VASP and EVL have an enhanced affinity for the profilin-2 isoform over profilin-1, whereas Mena shows no preference [29,46]. Additional differences between Ena/VASP members can be found within the PRR, which consists of profilin recruiting regions as well as high-affinity profilin binding sites, known as ‘loading sites’ [15]. Mena possesses four G-actin:profilin ‘loading sites’, whereas EVL and VASP each possess only one [15,43]. Single molecule assays demonstrated that EVL increases actin elongation to a rate of ∼20 subunits/s only in the presence of profilin-2, while VASP increases actin polymerization to ∼30 subunits/s with either profilin-1 or 2 [21,29]. Actin elongation rates correlate with the affinity of the GAB domain for actin monomers, which vary among Ena/VASP orthologues [17]. Tetramer mixing, therefore, may fine-tune the activity of Ena/VASP molecules by adjusting interactions with profilin and actin.

In a recent study [20], *Drosophila* Ena was shown to elongate actin filaments in a ‘tunable’ manner to promote the formation of filopodia. It was also shown that a single Ena tetramer could simultaneously elongate two filaments, although at a slower rate than when bound to the barbed end of a single filament. In the context of our results, these findings suggest that the kinetics of filopodia formation and dynamics could be influenced by the composition of Ena/VASP hetero-tetramers.

### Implications of mixed tetramer formation on the regulation of Ena/VASP function

Although Mena, VASP and EVL possess some overlapping functions, differences in their regulation and protein–protein interactions have been demonstrated. For example, VASP possesses multiple phosphorylation sites regulating its localization and activity that are absent from other family members. Additionally, Mena contains a unique sequence, termed the LERER region [1], that binds directly to the cytoplasmic tail of α5 integrin [28]. The interaction of Mena and α5 integrin influences the formation of fibrillar adhesions and the organization of extracellular matrix through the formation of fibronectin fibrils [28]. Mixed Mena:VASP tetramers have the potential to both bind α5 integrin and be regulated by paralogue-specific phosphorylation and interactions that influence their activity and localization.

VASP activity can be regulated by phosphorylation of residues that are not found in Mena or EVL. For instance, VASP phosphorylated T278 alters F-actin accumulation in cells [30], while Abl phosphorylation of VASP at Y39, another phosphorylation site absent from Mena and EVL, diminishes its localization at focal adhesions [47]. These additional sites allow for unique modes of regulation not observed with other Ena/VASP family members. However, how phosphorylation of VASP in mixed tetramers potentially alters the function and localization of these molecular complexes has not yet been evaluated.

Additional complexities in tetramer formation arise when one considers alternatively spliced isoforms of Ena/VASP proteins. EVL possesses a single alternatively included exon to produce the EVL-I isoform [46] and alternative splicing of Mena produces five distinct isoforms [1,40]. It has been demonstrated that alternatively spliced Ena/VASP proteins possess unique functions in cells. For example, cells expressing Mena^INV^ possess an enhanced sensitivity to low concentrations of EGF relative to cells expressing Mena^classic^ [27]. Another Mena isoform, Mena11a, has been shown to positively correlate with epithelial-like phenotypes [48,49] and negatively correlate with invasiveness [41,50]. How the function of Mena^INV^:Mena^classic^ or Mena11a:Mena^classic^ hetero-tetramers compare to Mena^INV^ or Mena11a homo-tetramers has not yet been evaluated.

Intriguingly, the role of Ena/VASP proteins in cancer progression and/or prognosis is not the same for all family members. For instance, increased expression of Mena is positively correlated with increased invasiveness of breast cancer cells and breast cancer grade [51], while EVL expression is negatively correlated with invasiveness and grade [29]. Our findings, in combination with the preferences of different Ena/VASP proteins for different profilin paralogues [15,29,46], suggest that distinct pools of Mena and EVL tetramers can exist within a single cell, and may therefore function independently of one another.

### LHCC allows for analysis of Ena/VASP homo-tetramers

Most studies examining Ena/VASP proteins have focused on one family member at a time. However, studies performed in cells expressing endogenous Ena/VASP proteins are complicated by the potential formation of mixed oligomers, which may modify the activities of the protein under investigation. Replacement of the native TD with a synthetic TD enables formation of pure oligomers containing only exogenously expressed Ena/VASP proteins. Thus, VASP^LHCC^ may provide a method to study the function of Ena/VASP homo-tetramers without concern for hetero-tetramerization with endogenous proteins.

Ena/VASP proteins are convergence points for regulation of actin polymerization by upstream signalling pathways [43]. Our findings strongly suggest that mixed tetramers provide another layer to this regulatory landscape. By blending or segregating Ena/VASP family members, the control of tetramer composition provides a mechanism to fine-tune actin polymerization in response to diverse signalling events.

## MATERIALS AND METHODS

### Antibodies

The following antibodies were used in this study and diluted 1:5000 for detection of western blots: monoclonal mouse αMena [23], polyclonal rabbit αMena (2197) [6], αVASP (2010) [6], αEVL polyclonal (1404) [46], monoclonal mouse αGFP (JL-8, Clontech), polyclonal rabbit αGFP (Invitrogen) and αFLAG (Clone M2, Sigma). For immunofluorescence, affinity-purified rabbit αLpd [33] was used at 1:400 and chicken αGFP (Ames Laboratory) and was diluted 1:500. Mouse αMena antibody was used for immunoprecipitation experiments of Mena and the rabbit αGFP antibody was used for the immunoprecipitation of EGFP-tagged proteins. Control whole molecule mouse and rabbit IgG was purchased from Jackson ImmunoResearch. Secondaries for western blots were purchased from Licor. 647-donkey αrabbit (Jackson Immunoresearch) and AlexaFluor 488 goat αchicken (Invitrogen) were used as secondaries for immunofluorescence. 647-Phalloidin (Invitrogen) and phalloidin CF405 conjugate (Biotium) were used to visualize F-actin.

### Plasmids

Sub-cloning and PCR using Phusion polymerase (NEB) were performed using standard techniques. pMSCV EGFP-VASP^LHCC^ was generated by replacing the TD of mouse VASP (residues 337–370) with residues 250–281 of synthetic pLI-GCN4 using PCR and restriction digests [36]. The following pCAX vectors were generated by subcloning: EGFP, EGFP-VASP^WT^, EGFP-VASP^LHCC^, EGFP-Mena and EGFP-EVL [1,2]. pCAX EGFP-EVH2(VASP) contains residues 221–375 of mouse VASP and pCAX EGFP-EVH2(Mena) contains residues 345–541 of mouse Mena.

pCAX FLAG-EGFP was created by fusing EGFP in-frame into pCDNA3-FLAG vector, and then subcloned into the pCAX vector to be used as the backbone for FLAG-tagged constructs. The following pCAX plasmids were cloned using the Gibson Assembly Master Mix (NEB, E2611): FLAG-VASP^LHCC^, FLAG-VASP^WT^, FLAG EVL and FLAG-Mena. The TD of mouse Mena (residues 501–541) was replaced with the TD of mouse EVL (residues 380–393) to create the chimeric EGFP-Mena^TD-EVL^ construct using Gibson assembly. All PCR primers for Gibson cloning were generated using NEBuilder (http://nebuilder.neb.com/) and were purchased from IDT.

### Cell culture and transfections

Rat2, MV^D7^ and derived cell lines were cultured as previously described [2]. MV^D7^ cells expressing EGFP-VASP^WT^ cells have been described [25], and MV^D7^ EGFP-VASP^LHCC^ and MV^D7^ EGFP-EVH2 (Mena) cells were generated using the same methodology. EGFP positive cells were sorted using FACS and were selected for matching EGFP expression levels.

MV^D7^ cells were transiently transfected with the indicated plasmids in the pCAX backbone using Amaxa Nucleofector Technology with cell nucleofection solution MEF-2 and program A-23, according to the manufacturer's recommendations (Lonza). Transfected cells were incubated at 32°C for 16–24 h prior to cells lysis and immunoprecipitation.

### Immunoprecipitation

10 cm dishes of cells were lysed in 250 μl IP lysis buffer [10% glycerol, 1% IGEPAL CA-630, 15 mM sodium pyrophosphate, 50 mM sodium fluoride, 50 mM TRIS (pH 7.5), 40 mM β-glycerophosphate, 200mM sodium chloride, 1mM sodium vanadate, 2 mM magnesium chloride and protease inhibitor tablet without EDTA (Roche)]. Lysates were incubated on ice for 10 min, and then centrifuged at 20 000 ***g*** for 10 min at 4°C on a table-top centrifuge. For EGFP, Mena and VASP IPs, lysates were precleared with Dynabeads Protein G (Life Technologies) for 1 h, then incubated with the indicated antibody for 1 h at 4°C and finally captured with BSA-blocked Dynabeads for 1 h. For FLAG IPs, clarified lysates were incubated with BSA-blocked Anti-DYKDDDK Magnetic Beads (Clontech) for 2 h. Beads were washed three times in lysis buffer, and proteins were eluted in 4X Laemmli sample buffer [3% SDS, 1% Glycerol, 1.5% β-mercaptoethanol, 50mM Tris (pH 6.8), 100mM DTT and 0.1% Bromophenol Blue].

### Western blot analysis and quantification

Lysates and immunoprecipitated proteins were separated on SDS/PAGE gels and transferred on to nitrocellulose membranes (Bio-Rad) for 2 h at 80 V in transfer buffer supplemented with 10% methanol. Membranes were blocked with Odyssey Blocking Buffer (LiCor) and probed with the indicated antibodies. Membranes were washed with PBST and membrane-bound proteins were detected by infrared (LiCor) imaging. Images were recorded as TIFF files for quantification. Band intensities were measured using FIJI [52]. For presentation, original TIFF files were inverted and contrast enhanced using Adobe Photoshop. Merged colour images were pseudocoloured using Adobe Photoshop.

For crosslinking experiments, samples were run on a SDS/6% PAGE and transferred on to a PVDF membrane for 3 h at 90 V in transfer buffer supplemented with 20% methanol. The membrane was blocked in 5% milk in PBST and EGFP-EVH2 protein was detected on film using the mouse αGFP antibody and Lumi-Light western blotting substrate (Roche).

### Random mixing model

Assuming random mixing of Mena/VASP members within a cell that expresses a particular ratio of two members, the distribution of tetramers should follow a binomial distribution such that:

$$\;F_{k}=\left(\begin{array}{c} 4\\ k \end{array}\right)\;p^{k}{\left(1-p\right)}^{4-k}$$

where *F_k_* is the fraction of tetramers with *k* members of VASP, and *p* is the fraction of Mena/VASP protein that is VASP. Co-immunoprecipitation experiments were therefore simulated by removal of tetramers lacking Mena, and comparison to the overall population.

### Immunofluorescence

MV^D7^ cells stably expressing constructs were plated on acid washed coverslips coated with fibronectin (10 μg/ml, Sigma). Cells were fixed with 4% paraformaldehyde (PFA) in PHEM buffer [60 mM PIPES (pH 7.0), 25 mM HEPES (pH 7.0), 10 mM EGTA, 2 mM MgCl_2_, 0.12 M sucrose] for 15 min at room temperature, and permeabilized with 0.2% Triton X-100 in PBS for 10 min. Coverslips were rinsed with PBS, then blocked with 10% donkey serum for 1 h, followed by incubation with primary antibodies diluted in 1% donkey serum. Coverslips were washed three times with PBS, and then incubated with fluorescent phalloidin and appropriate fluorescent secondary antibodies in 1% donkey serum. Coverslips were washed three times with PBS and mounted on glass slides using Fluoroshield with DABCO (Sigma).

### Cell spreading assay and CD treatment

MV^D7^ cells stably expressing EGFP-VASP constructs were trypsinized, resuspended in media and re-plated on fibronectin-coated coverslips (10 μg/ml). Cells were allowed to spread for 20 min followed by fixation with 4% PFA in PHEM buffer for 15 min at room temperature. Coverslips were then prepared for imaging as above.

Cytochalasin D (CD) (Sigma–Aldrich) was dissolved in DMSO for stock solutions of 5 mM and stored at -20°C. MV^D7^ cells were plated on fibronectin-coated (10 μg/ml) coverslips for 4 h, followed by incubation with media containing diluted CD for 30 min, then fixed and prepared for imaging as described above.

### Imaging

Cells were imaged on a DeltaVision microscope (Applied Precision) using either a 60× 1.3 NA Plan-Apochromat or a 40× 1.3NA UPlan-FL N objective lens (Olympus). z-stacks were acquired with a CoolSNAP HQ camera (Photometrics) and SoftWoRx acquisition software (Applied Precision). Images were deconvolved using Deltavision SoftWoRx software and objective specific point spread function. Projections of three z-stacks were made using FIJI.

### Quantification of VASP leading edge localization

Masks of the cell area were created by thresholding phalloidin images and total GFP intensity was determined by measuring the intensity of the appropriate channel in the masked area. The ImageJ Edge ratio plugin [53] (http://www.unc.edu/∼cail/code/EdgeRatio.txt) was used to quantify the GFP intensity at fixed distances from the cell edge of Lpd-positive regions. The intensity value was normalized to the total cell intensity to compensate for variations in GFP expression. The mean values of the normalized intensity were plotted with error bars representing S.E.M. Two-way ANOVA was performed with Tukey Multiple Comparison post-test using GraphPad Prism version 6.04 for Mac, GraphPad Software.

### Cross-linking of EGFP-EVH2 (Mena) in lysates

MV^D7^ cells stably expressing EGFP-EVH2 (Mena) were lysed in a modified IP lysis buffer [10% glycerol, 1% IGEPAL CA-630, 15 mM sodium pyrophosphate, 50 mM sodium fluoride, 50 mM MES (pH 6.0), 40 mM β-glycerophosphate, 500mM sodium chloride, 2mM magnesium chloride and protease inhibitor tablet without EDTA (Roche)]. Following a 10-min incubation on ice and centrifugation (14,000 rpm, 10 min, 4°C), EDC (final concentration 100mM, Pierce) and NHS-ester (final concentration 10mM, Thermo-Scientific) were added to the lysates and incubated at room temperature for 1 h. Untreated lysates were incubated in lysis buffer. Cross-linking was quenched with the addition of β-mercaptoethanol (final concentration 20 mM, Sigma) and 4× sample buffer.

## Abbreviations

co-IP: co-immunoprecipitate
Ena/VASP: enabled/vasodilator-stimulated phosphoprotein
EVH: Ena/VASP homology
FAB: F-actin binding
F-actin: filamentous actin
GAB: G-actin binding
G-actin: globular/monomeric actin
Lpd: lamellipodin
PRR: proline-rich region
TD: tetramerization domain
